# Supplementary material for: Stratification of TAD boundaries reveals preferential insulation of super-enhancers by strong boundaries
Source: Nat Commun. 2018 Feb 7;9:542. doi: 10.1038/s41467-018-03017-1 (PMC5803259; doi:10.1038/s41467-018-03017-1)
Supplement: Supplementary file 1 — Supplementary Information [file 41467_2018_3017_MOESM1_ESM.pdf]

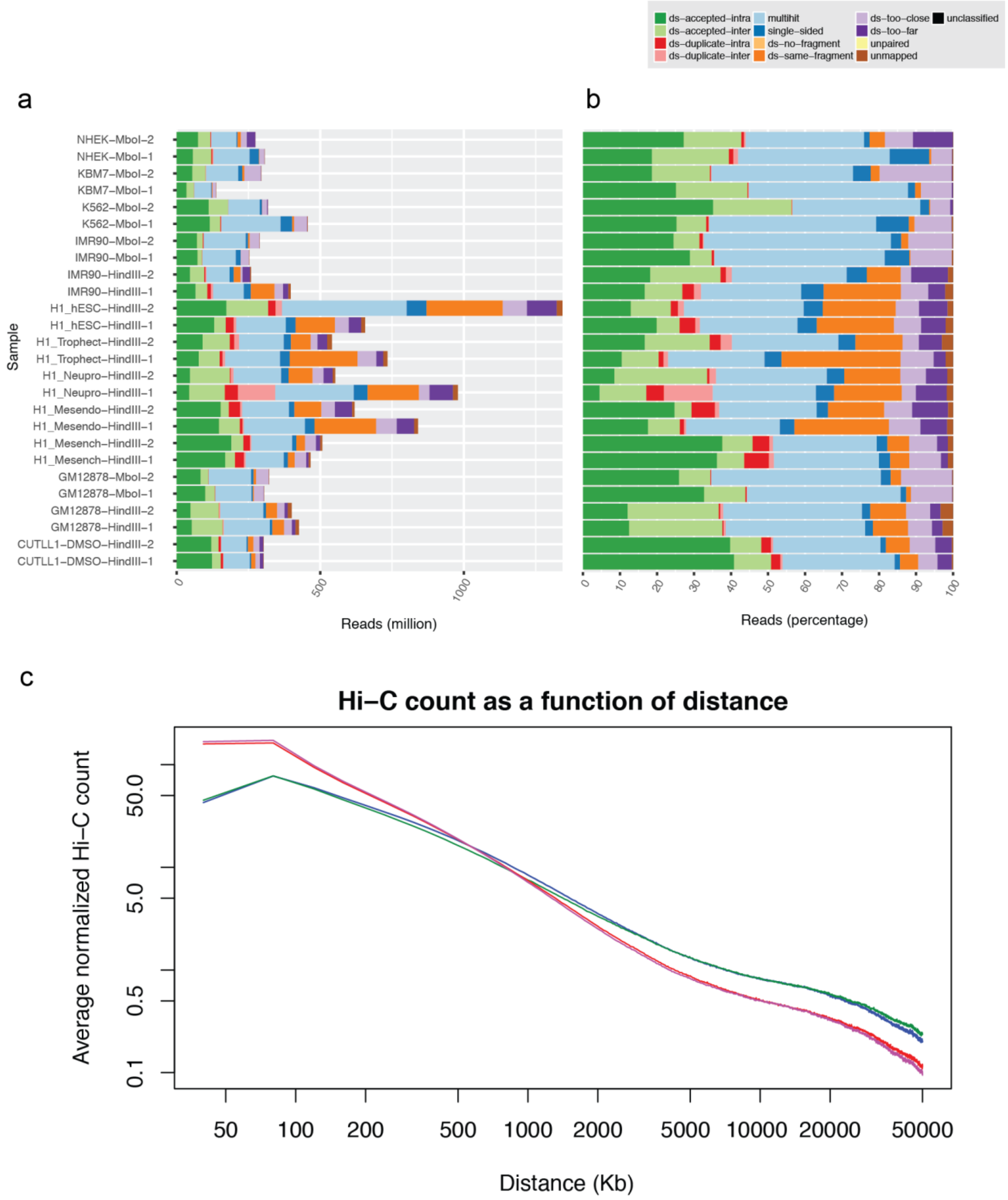

Supplementary Figure 1. Quality assessment of Hi-C datasets. (a) Counts of Hi-C read pairs in various read categories: dark and light green indicate read pairs that were not designated as artifacts and can be used in downstream analyses; duplicate read pairs (ds-duplicate-intra and ds-duplicate-inter) are marked red and pink respectively; non-uniquely mappable (multihit) are show in in light blue, single-end mappable (single-sided) in dark blue and unmapped reads (unmapped) in dark purple; self-ligation products (ds-same-fragment) are marked orange; reads mapping too far or too close (ds-too-far or ds-too-close) from/to restriction sites are shown in light purple and orange respectively, **(b)** Percentages of Hi-C reads in each category, **(c)** Average scaled Hi-C read pair count as a function of distance between interacting loci: different colors represent different replicates of the IMR90 samples (two replicates for each enzyme).

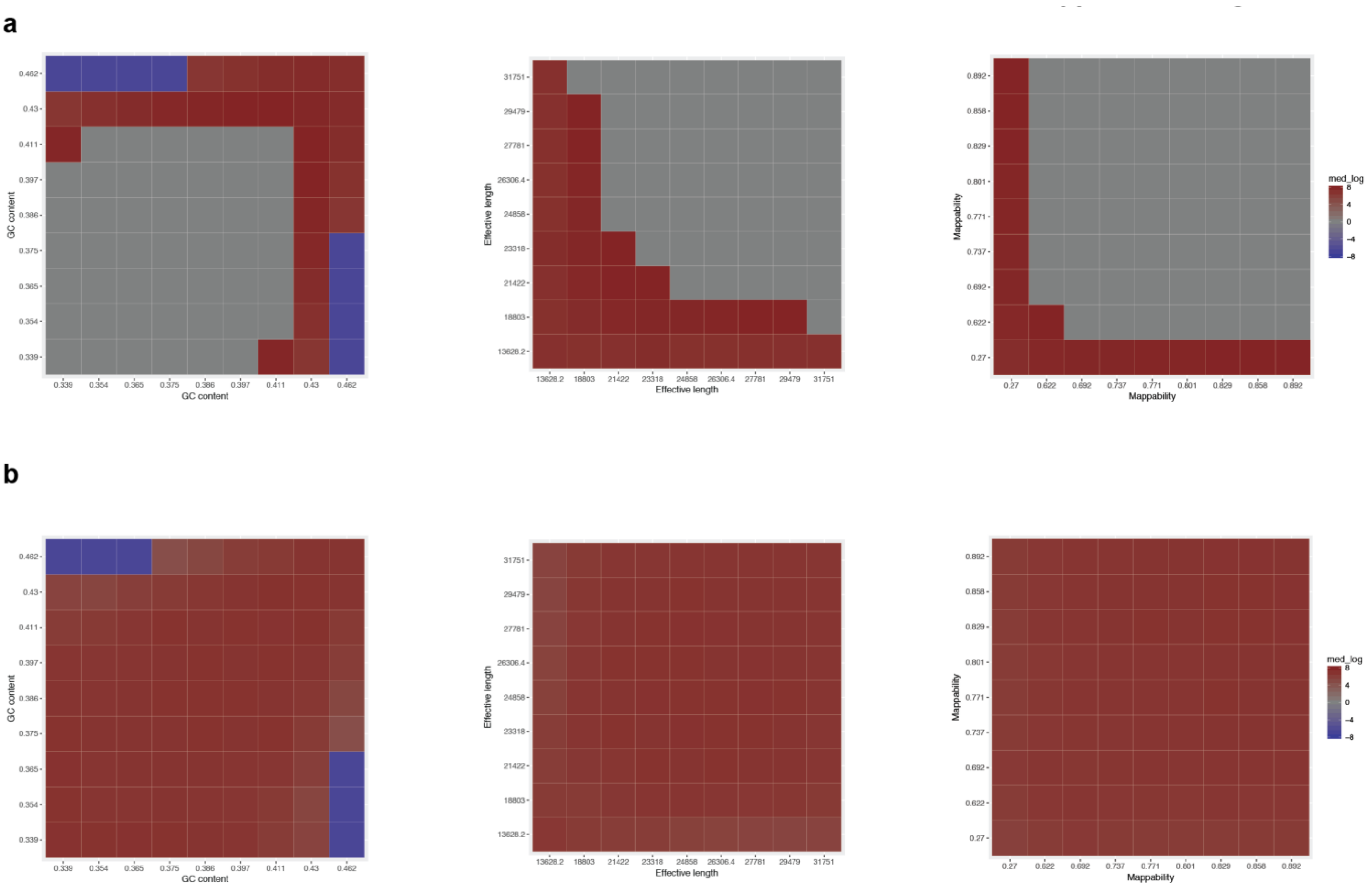

Supplementary Figure 2. Scaling method corrects for enzyme-specific biases. (a) Unprocessed Hi-C matrices exhibit enzyme-specific biases related to the GC content, effective length and mappability of the interacting bins, **(b)** In contrast, our scaling method corrects for all three enzyme-specific biases.

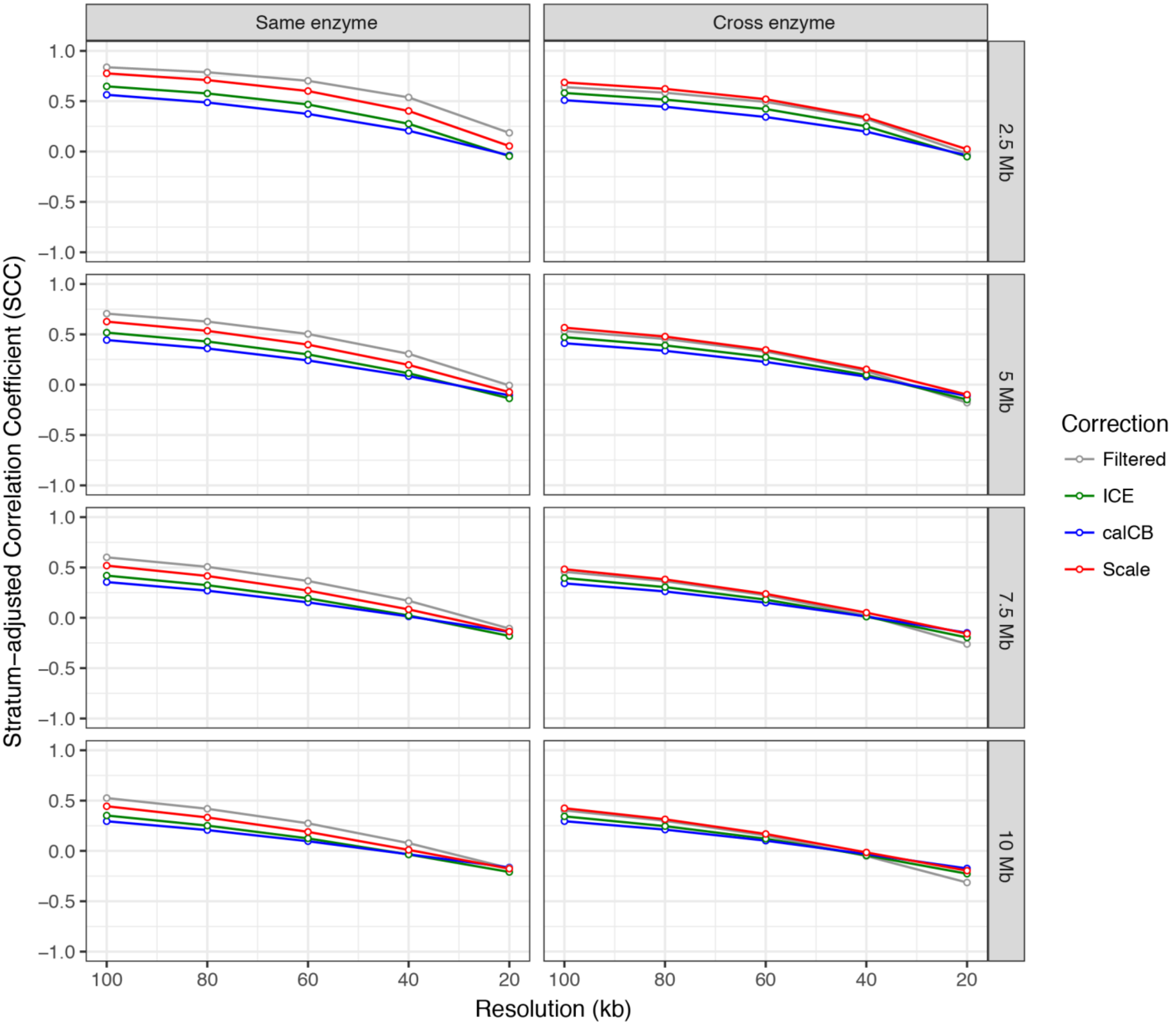

Supplementary Figure 3. Reproducibility of Hi-C contact matrices (*low sequencing depth = 40 million intrachromosomal read pairs*). Comparison of Hi-C contact matrices between biological replicates generated from Hi-C library using the same or different restriction enzymes; Hi-C matrices were estimated using four methods (naïve filtering, iterative correction, calCB and simple scaling); assessment was performed using stratum-adjusted correlation coefficient at resolutions ranging from 100kb to 20kb and maximum distances of 2.5Mb, 5Mb, 7.5Mb and 10Mb between interacting pairs.

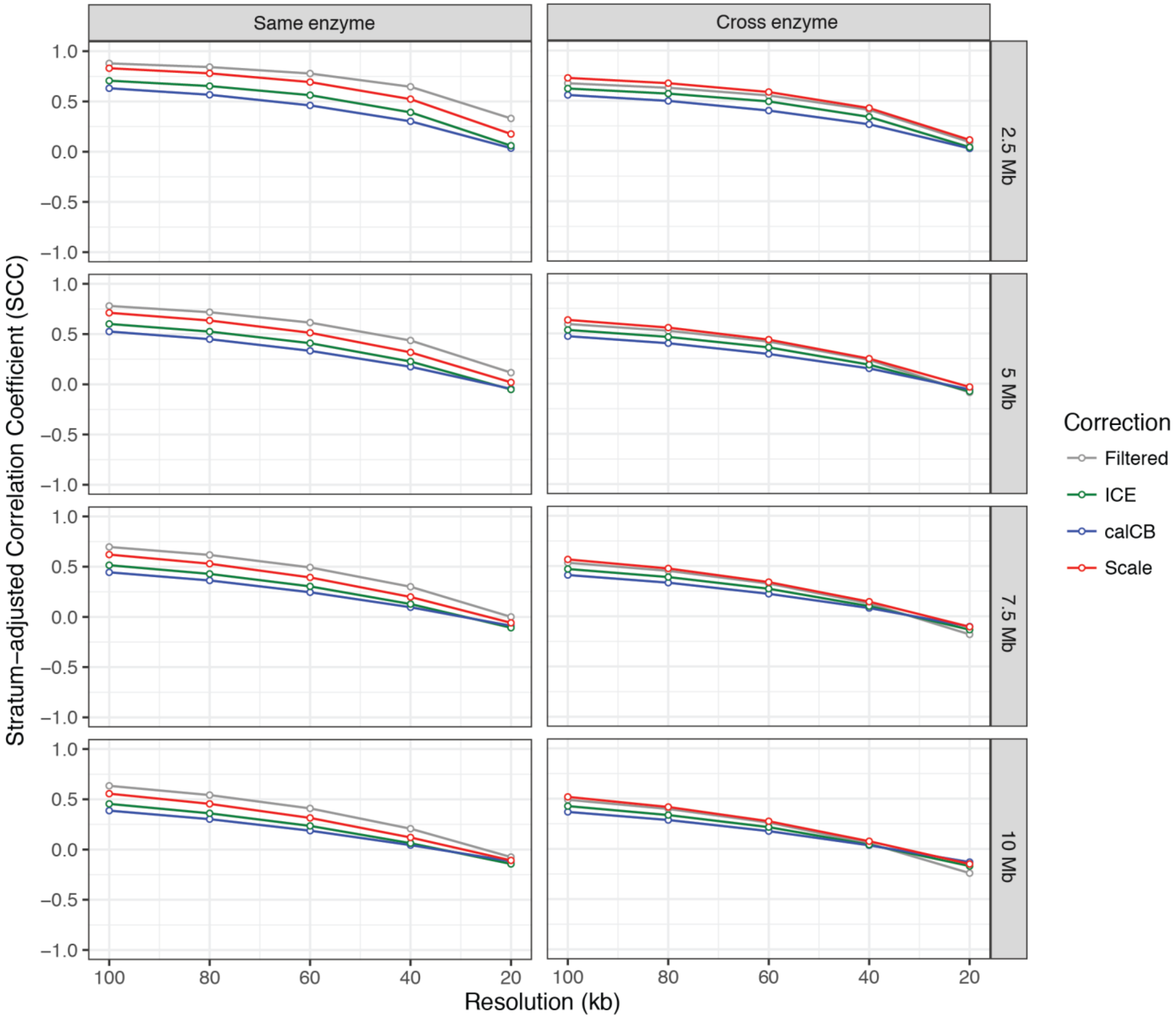

Supplementary Figure 4. Reproducibility of Hi-C contact matrices (*high sequencing depth = 80 million intrachromosomal read pairs*). Comparison of Hi-C contact matrices between biological replicates generated from Hi-C library using the same or different restriction enzymes; Hi-C matrices were estimated using four methods (naïve filtering, iterative correction, calCB and simple scaling); assessment was performed using stratum-adjusted correlation coefficient at resolutions ranging from 100kb to 20kb and maximum distances of 2.5Mb, 5Mb, 7.5Mb and 10Mb between interacting pairs.

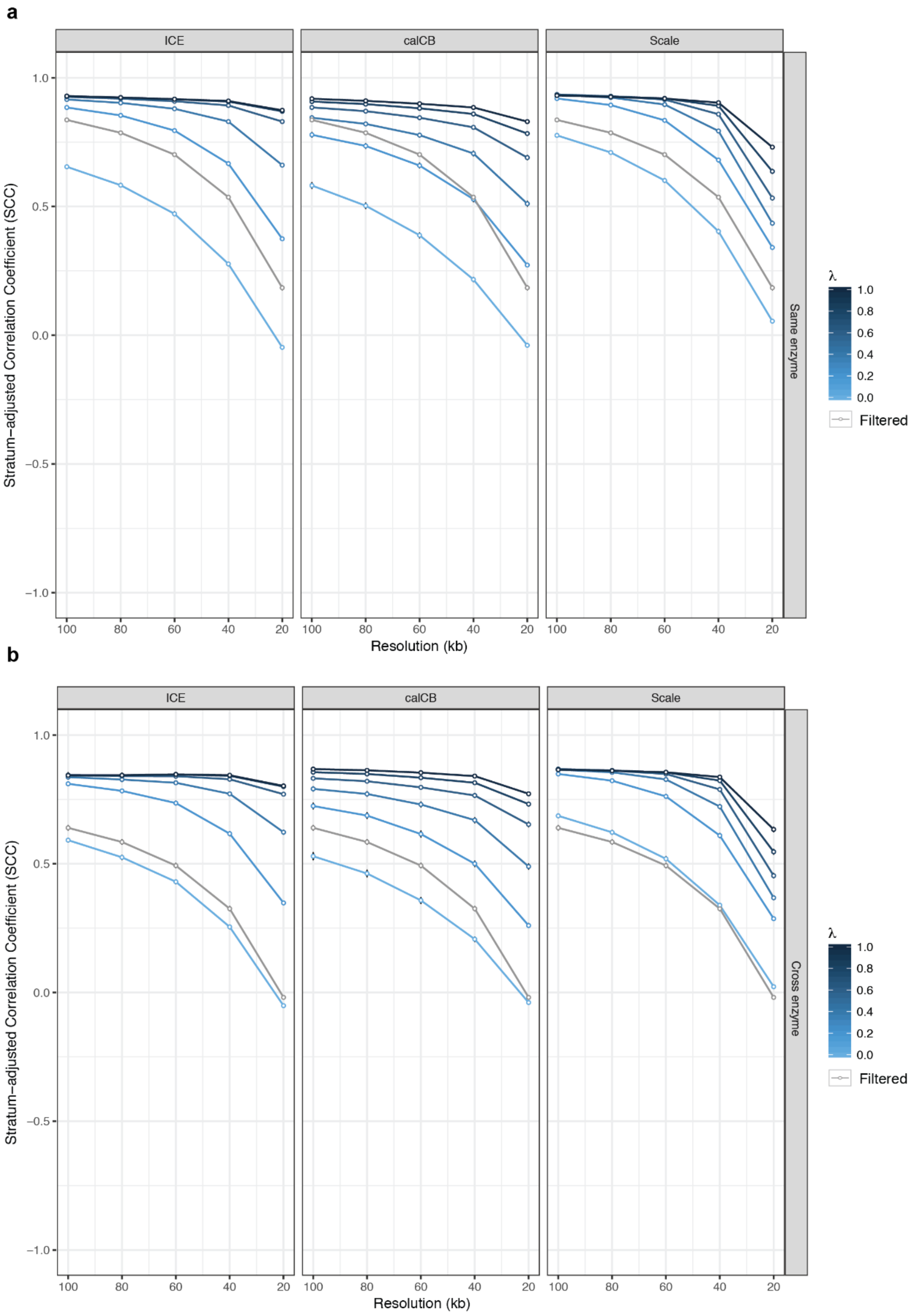

Supplementary Figure 5. Fused two-dimensional lasso improves reproducibility of Hi-C contact matrices (*low sequencing depth = 40 million intrachromosomal read pairs*). (a) Stratum-adjusted correlation coefficient values are improved by increasing the value of fused lasso parameter λ for matrices estimated with ICE, calCB and our simple scaling method (same enzyme), **(b)** Stratum-adjusted correlation coefficient values are improved by increasing the value of fused lasso parameter λ for matrices estimated with ICE, calCB and our simple scaling method (cross enzyme). As a baseline control, stratum-adjusted correlation coefficients of Hi-C contact matrices generated by the naïve filtering method are marked by the gray line in each panel.

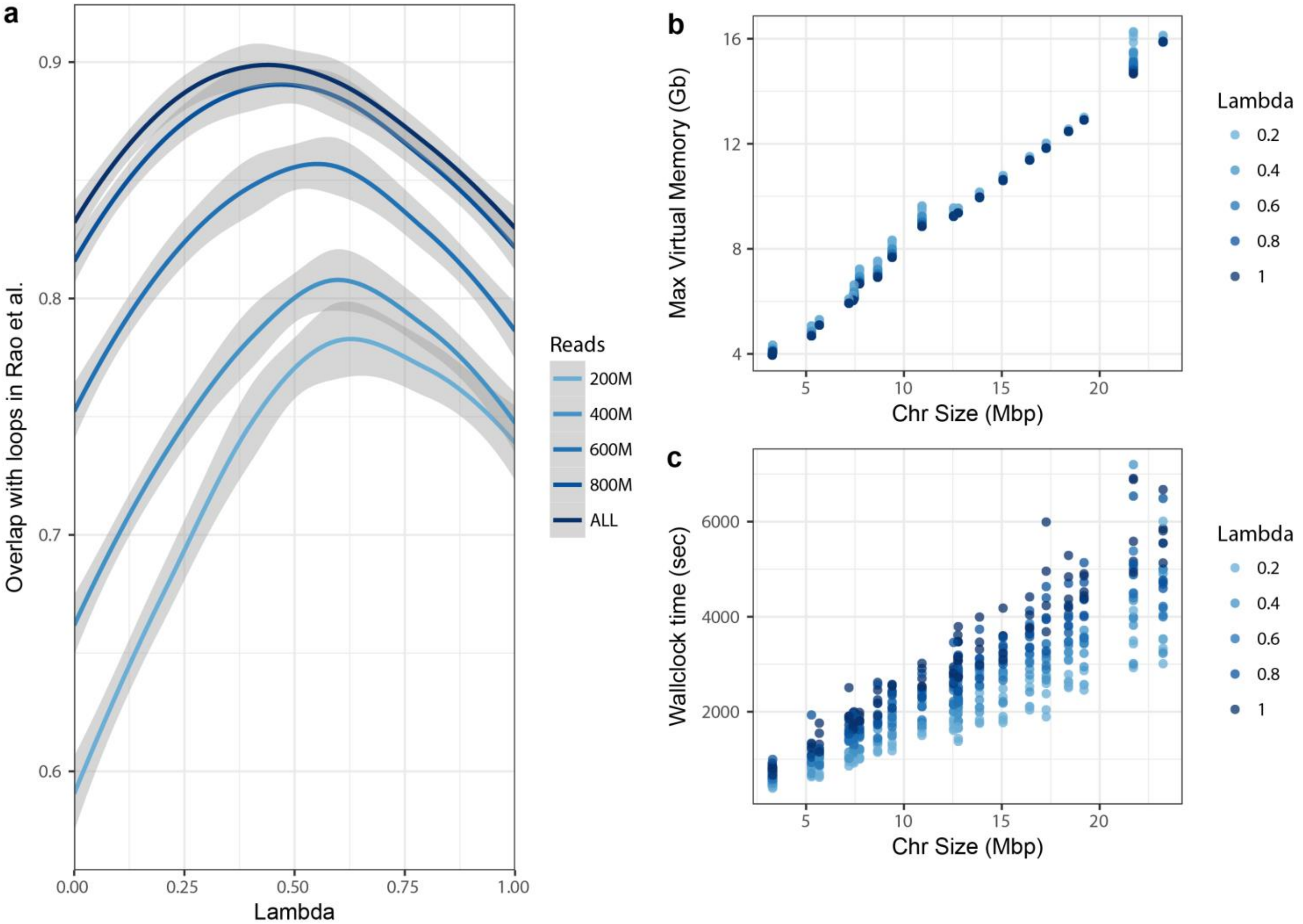

Supplementary Figure 6. Application of fused 2D lasso at fine resolutions. (a) Fraction of loops reported in Rao *et al.*[30] at 5kb resolution that are within the top 10% of DNA-DNA interactions obtained from the application of the fused lasso algorithm (for different λ values and varying sequencing depth) on the ICE-corrected Hi-C matrices (the line is calculated using loess regression and the shaded area is the 95% confidence region), **(b)** Maximum memory requirements of the fused lasso algorithm as a function of chromosome size, **(c)** CPU usage of the fused lasso algorithm as a function of chromosome size.

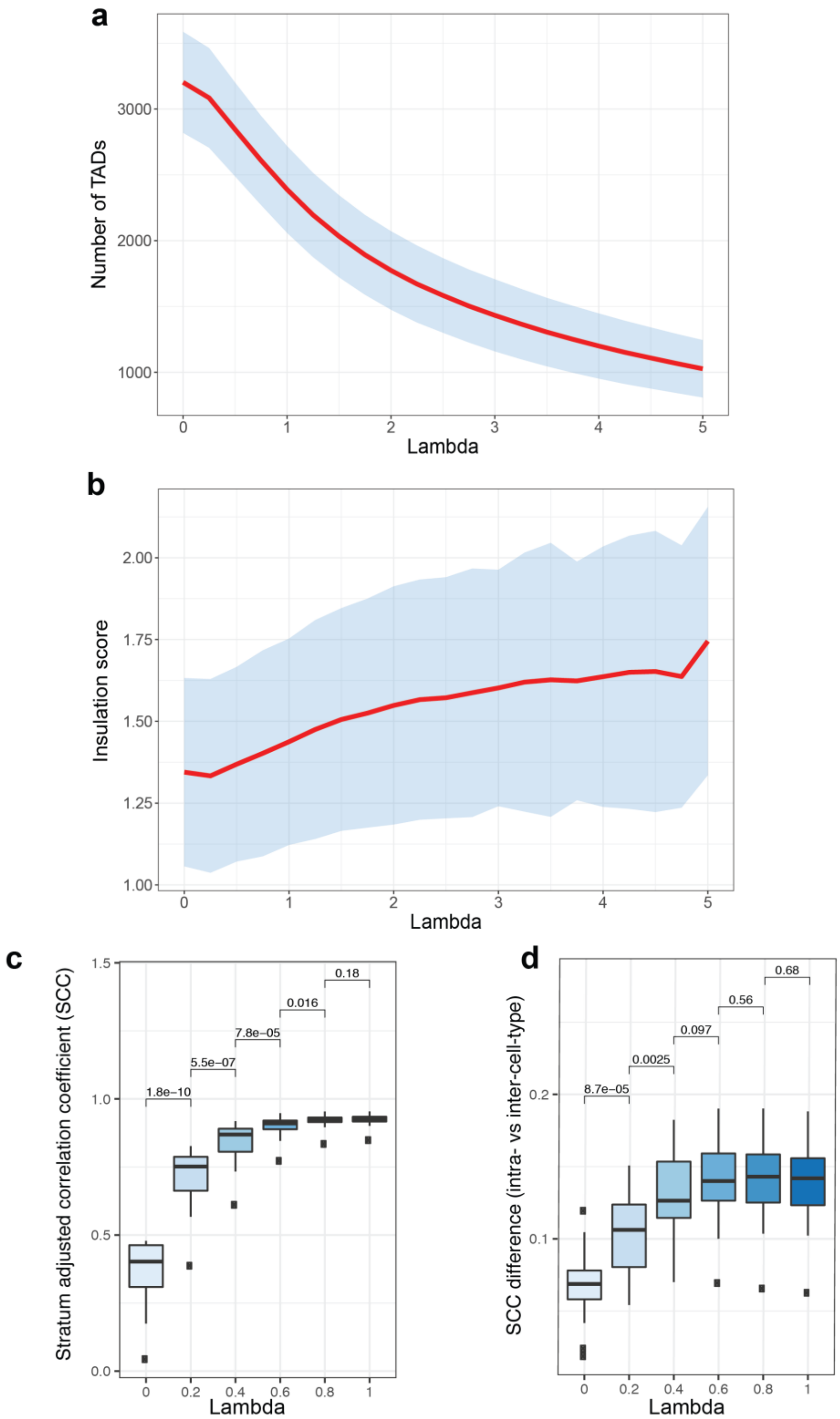

Supplementary Figure 7. Association of *λ*-boundaries with insulation score. (a) Number of TADs for *λ* values ranging from 0 to 5 (the line represents the mean and the shaded area one standard deviation from the mean), **(b)** TAD boundaries lost at higher values of parameter *λ* are associated with higher TAD insulation scores (the line represents the mean and the shaded area one standard deviation from the mean), **(c)** Calculation of optimal *λ* by comparing the statistical significance (measured by a two-sided Wilcoxon rank sum test) of the improvement of reproducibility between successive *λ* values (each dot in the boxplots represents a different chromosome), **(d)** Calculation of optimal *λ* by comparing the statistical significance (measured by a two-sided Wilcoxon rank sum test) of the improvement of the intra- vs inter-cell-type similarity between successive *λ* values (each dot in the boxplots represents a different chromosome).

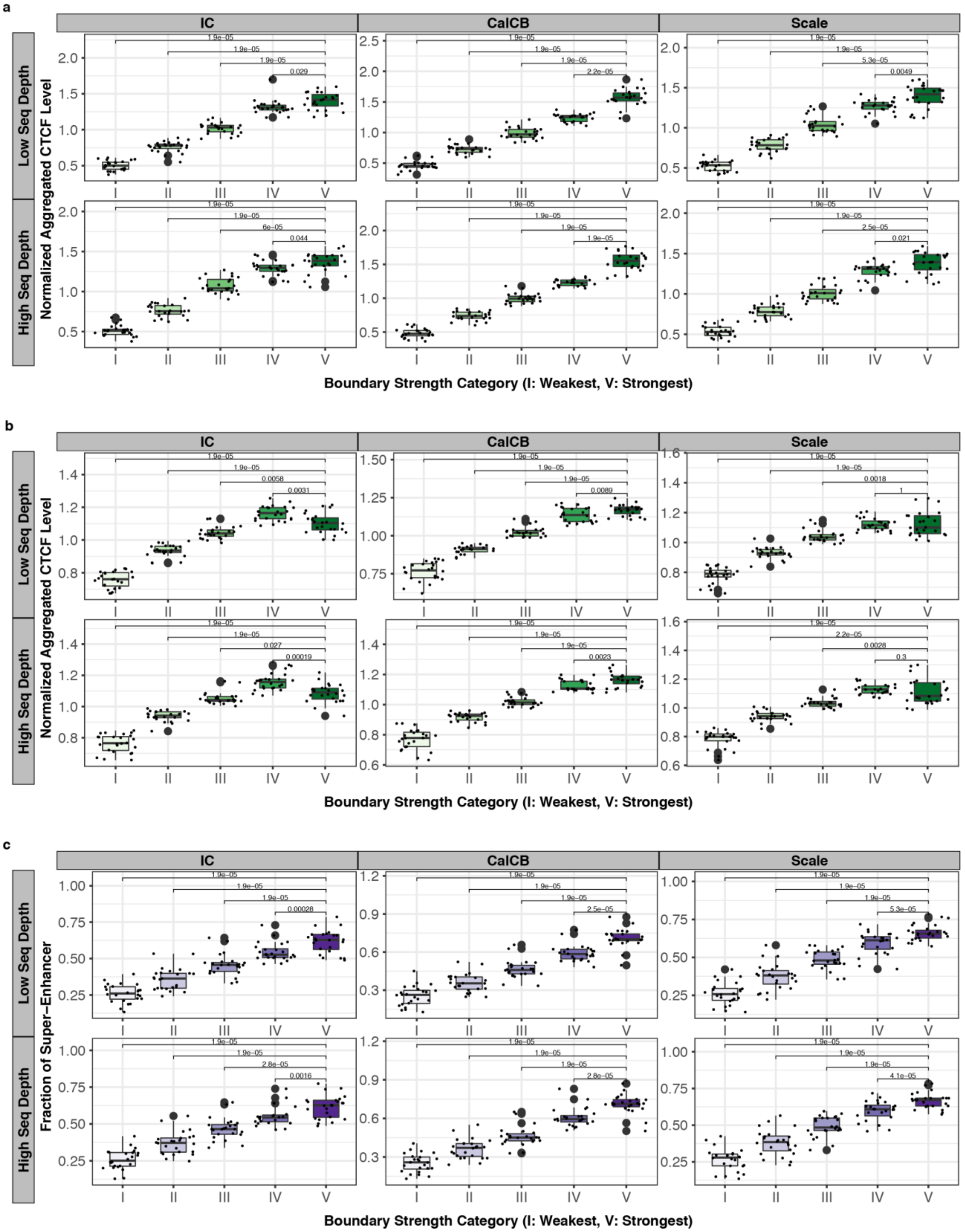

Supplementary Figure 8. Robustness analysis of the classification and characterization of TAD boundaries according to the "ratio" insulation score (*low and high sequencing depths*). Independent of Hi-C matrix "correction" method and sequencing depth, TAD boundary "ratio" insulation scores are associated with: **(a)** CTCF levels at TSSs, **(b)** CTCF levels (both TSS and non-TSS), **(c)** proximity to super-enhancer elements. All statistical tests are paired two-sided Wilcoxon rank sum tests between distributions defined across samples (each sample is a dot in the boxplots).

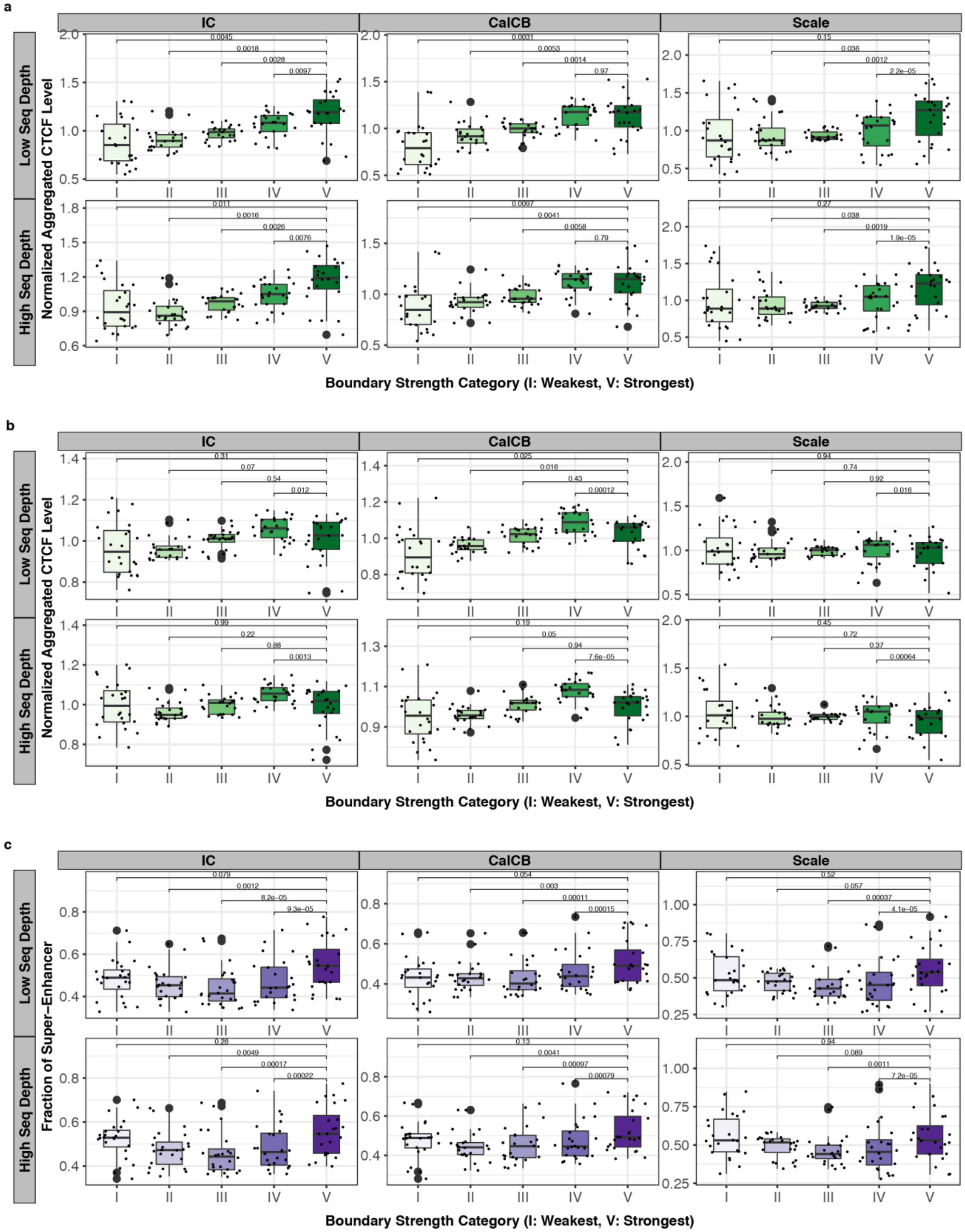

Supplementary Figure 9. Robustness analysis of the classification and characterization of TAD boundaries according to the “crane” insulation score (*low and high sequencing depths*). Independent of Hi-C matrix “correction” method and sequencing depth (but not as significantly as “ratio” insulation scores) TAD boundary “crane” insulation scores are (weakly) associated with: **(a)** CTCF levels at TSSs, **(b)** CTCF levels (both TSS and non-TSS), **(c)** proximity to super-enhancer elements. All statistical tests are paired two-sided Wilcoxon rank sum tests between distributions defined across samples (each sample is a dot in the boxplots).

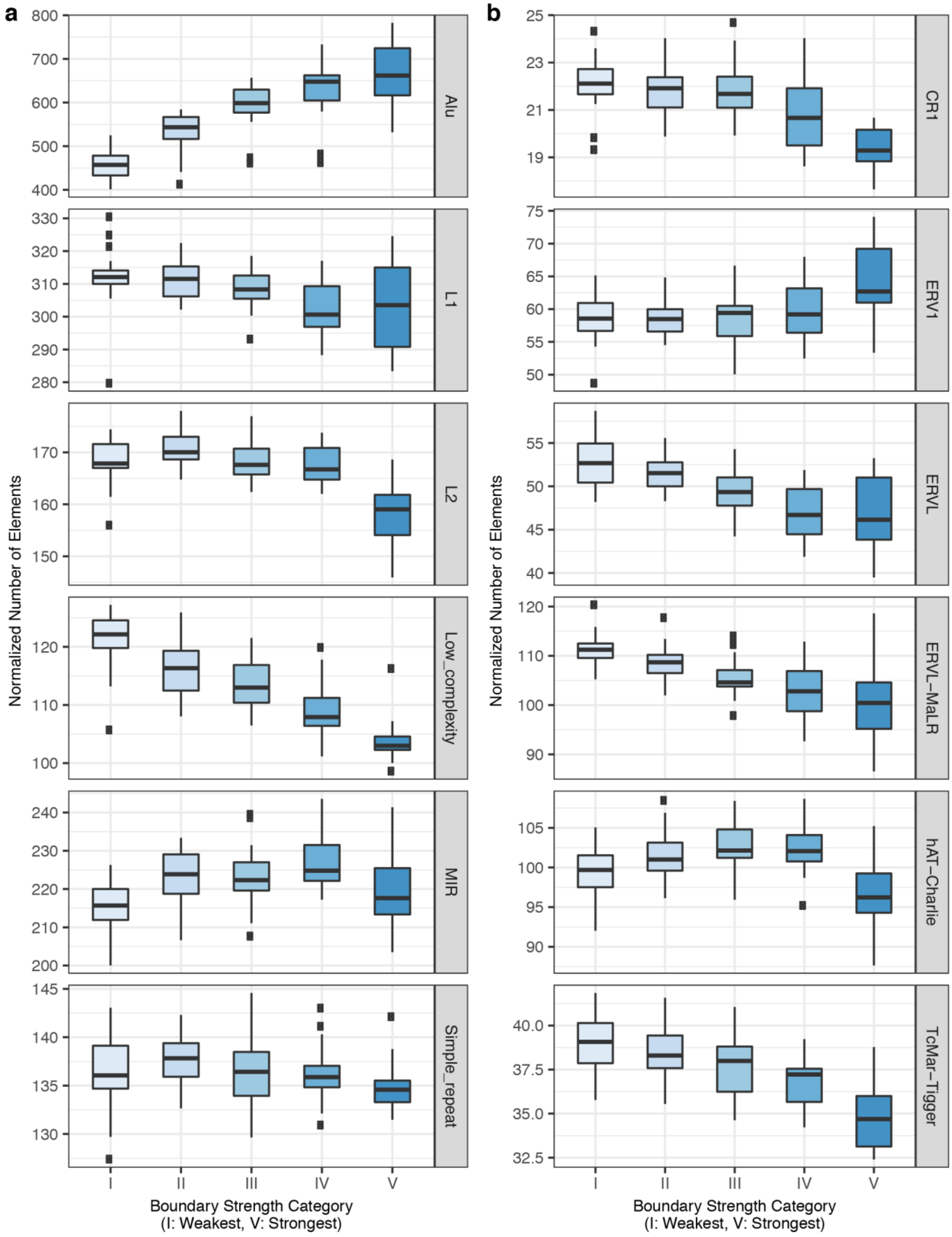

Supplementary Figure 10. Association of repeat elements with TAD insulation scores. Normalized numbers of repeat elements in proximity to boundaries of certain boundary strength. The distributions represented by the boxplots are across samples and replicates. Darker blue in the blue colour gradient denotes higher boundary strength.

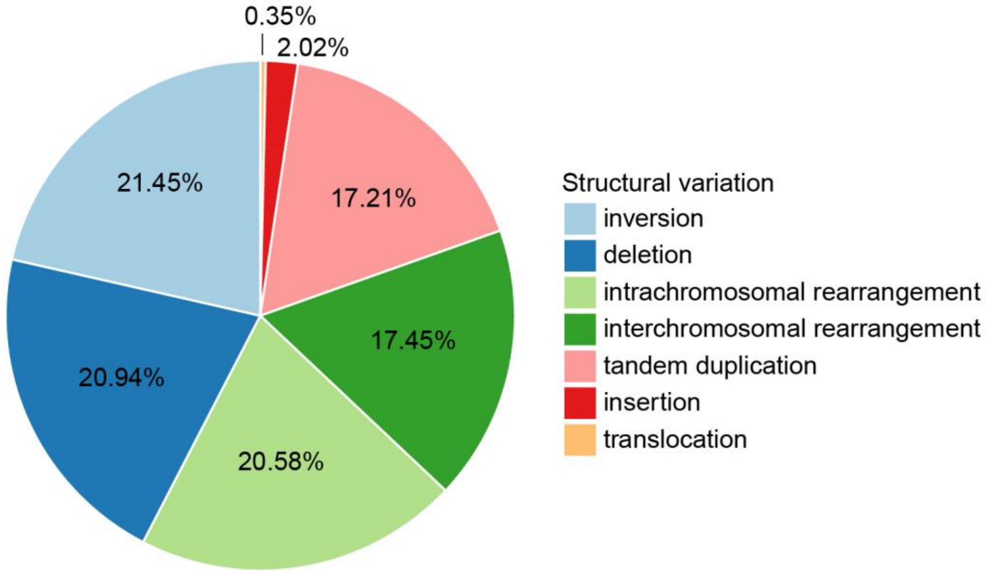

Supplementary Figure 11. Distribution of somatic structural alterations in the ICGC database.

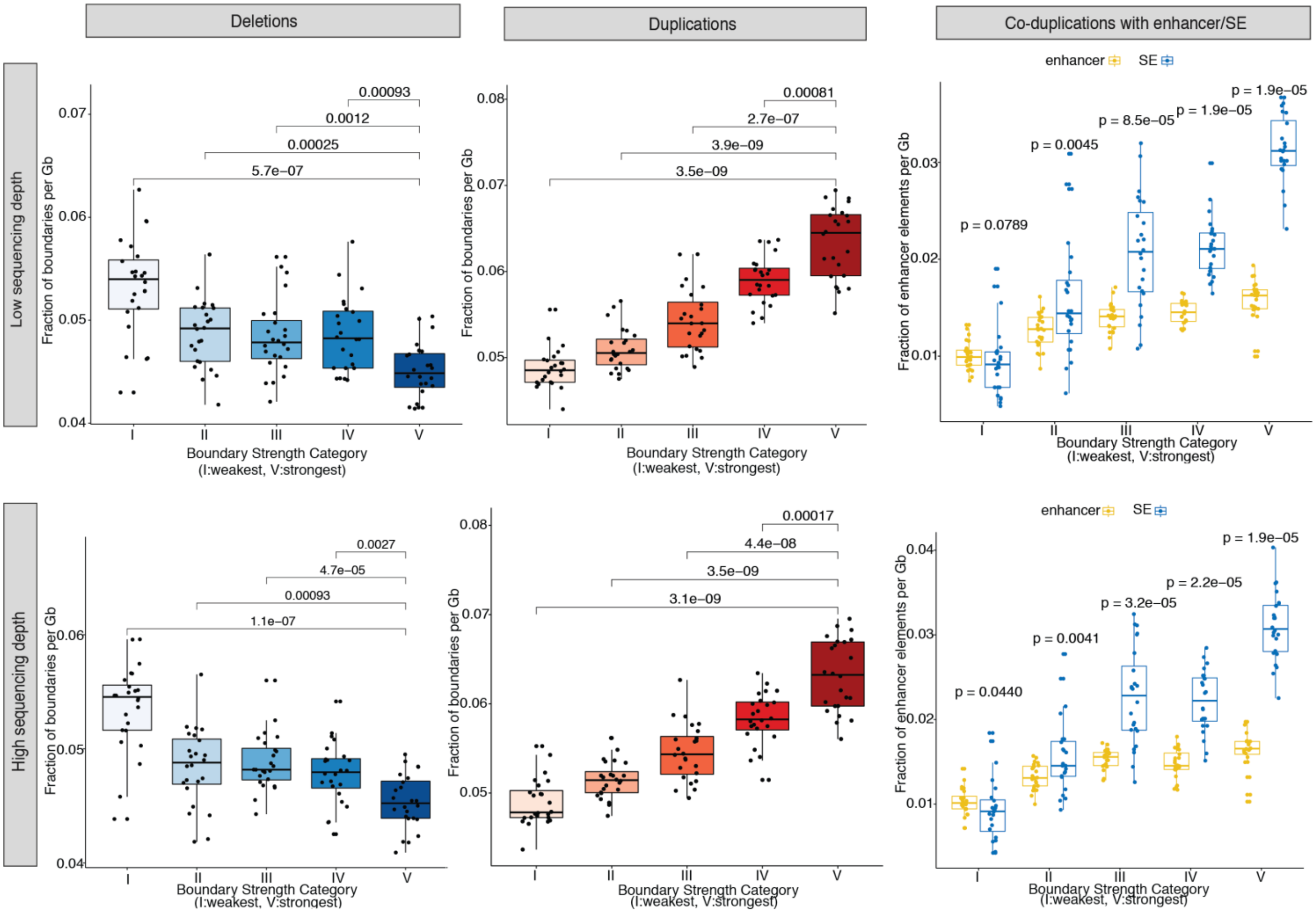

Supplementary Figure 12. Robustness analysis of the association of strong vs weak TAD boundaries with deletions and tandem duplications in cancer patients (*low and high sequencing depths*). Results presented in Error! Reference source not found.**a-c** are consistent, independent of sequencing depth: **(a)** Classification of focally deleted boundaries in cancer according to their strength, **(b)** Classification of focally duplicated boundaries in cancer according to their strength, **(c)** Co-duplications of TAD boundaries with regular enhancers and super-enhancers in cancer. All statistical tests are paired two-sided Wilcoxon rank sum tests between distributions defined across samples (each sample is a dot in the boxplots).
