# Supplementary material for: Stratification of TAD boundaries reveals preferential insulation of super-enhancers by strong boundaries
Source: Nat Commun. 2018 Feb 7;9:542. doi: 10.1038/s41467-018-03017-1 (PMC5803259; doi:10.1038/s41467-018-03017-1)
Supplement: Supplementary file 3 — Description of Additional Supplementary File [file 41467_2018_3017_MOESM3_ESM.pdf]

Description of Additional Supplementary File

File Name: Supplementary Data 1
Description: Description and accession numbers of Hi-C, CTCF and H3K27ac datasets.
